# X-ray microtomography analysis of soil pore structure dynamics under wetting and drying cycles

**DOI:** 10.1016/j.geoderma.2019.114103

**Published:** 2020-03-15

**Authors:** Luiz F. Pires, André C. Auler, Waldir L. Roque, Sacha J. Mooney

**Affiliations:** a Laboratory of Physics Applied to Soils and Environmental Sciences, Department of Physics, State University of Ponta Grossa (UEPG), 84.030-900, Ponta Grossa, PR, Brazil; b Department of Soils and Agricultural Engineering, Federal University of Paraná, 80.035-050, Curitiba, PR, Brazil; c Petroleum Engineering Modelling Laboratory, Department of Scientific Computation, Federal University of Paraíba, 58.051-900, João Pessoa, PB, Brazil; d Division of Agricultural and Environmental Sciences, School of Biosciences, University of Nottingham, Sutton Bonington Campus, Leicestershire LE12 5RD, UK

**Keywords:** Soil structure, Pore shape, Pore size distribution, Soil micromorphology, 3D image analysis

## Abstract

The soil water retention curve is one of the most important properties used to predict the amount of water available to plants, pore size distribution and hydraulic conductivity, as well as knowledge for drainage and irrigation modeling. Depending on the method of measurement adopted, the water retention curve can involve the application of several wetting and drying (W-D) cycles to a soil sample. The method assumes soil pore structure is constant throughout however most of the time soil structure is dynamic and subjected to change when submitted to continuous W-D. Consequently, the pore size distribution, as well as other soil morphological properties can be affected. With this in mind, high resolution X-ray Computed micro-Tomography was utilized to evaluate changes in the soil pore architecture following W-D cycles during the procedure of the water retention curve evaluation. Two different soil sample volumes were analyzed: ROI_W_ (whole sample) and ROI_HC_ (the region close to the bottom of the sample). The second region was selected due to its proximity to the hydraulic contact of the soil with the water retention curve measurement apparatus. Samples were submitted to the following W-D treatments: 0, 6 and 12 W-D. Results indicated the soil changed its porous architecture after W-D cycles. The image-derived porosity did not show differences after W-D cycles for ROI_W_; while for ROI_HC_ it increased porosity. The porosity was also lower in ROI_HC_ in comparison to ROI_W._ Pore connectivity improved after W-D cycles for ROI_HC_, but not for ROI_W_. W-D cycles induced more aligned pores for both ROIs as observed by the tortuosity results. Pore shape showed changes mainly for ROI_W_ for the equant and triaxial shaped pores; while pore size was significantly influenced by the W-D cycles. Soil water retention curve measurements showed that W-D cycles can affect water retention evaluation and that the changes in the soil morphological properties can play an important role in it.

## Highlights

• µCT allowed quantifying morphological changes in the region of the sample close to the hydraulic contact.
• 3D images permitted detailed analysis of the pore shape and size distribution.
• Tortuosity and pore connectivity was affected by wetting and drying cycles.
• Soil water retention curve was influenced by wetting and drying cycles.

## 1 Introduction

The soil water retention curve is a very important soil physical-hydraulic property, expressed by the relationship between the pressure head of the soil and its water content (Klute, 1986). The soil water retention curve can be used to evaluate different parameters such as the amount of water available to the plants, field capacity, permanent wilting point, pore size distribution, etc. (Hillel, 2004, Radcliffe and Simùnek, 2010). The methods used to determine this property typically require equipment such as suction tables, pressure chambers, gamma-ray sources and tensiometers (Smagin, 2012, Braudeau et al., 2014).

The relation between the pressure head and soil water content can be obtained in two ways, desorption (drying) and sorption (wetting). Continuous curves are obtained in both methods, but in general, they are not identical due to hysteresis (Hillel, 2004). The soil water retention curve determination involves the measurement of a series of equilibria of the water in the soil sample at known pressure heads. Depending on the experimental procedure chosen samples can be submitted to several wetting and drying (W-D) cycles (Moraes et al., 1993, Kong et al., 2018, Reis et al., 2019).

Moraes et al. (1993) presented an analysis of methodological problems during evaluation of the water retention curve examining 250 curves obtained through suction tables and pressure chambers. They verified 43% of the samples did not show consistent results and pointed out that effective hydraulic contact is crucial for the evaluation of representative soil water retention curves. Additionally, soil structure changes caused by the application of W-D cycles can affect the water retention curve quality (Bacchi et al., 1998, Pires et al., 2008a, Liu et al., 2012, Sayem and Kong, 2016, Zhang et al., 2017, Kong et al., 2018). The rearrangement of particles inside the soil matrix affecting soil resistance, particle cohesion, internal friction, clay dispersion, aggregate size and stability can be induced by the application of W-D (Rajaram and Erbach, 1999).

Thus, possible changes in soil pore structure in different regions of the soil sample could help to explain differences in water retention curve when samples are submitted to several W-D (Hussein and Adey, 1998, Pires et al., 2005, Pires et al., 2008a, Zhang et al., 2017). One part of the soil sample that is of particular interest is the region close to the hydraulic contact to the porous plate or sandbox. It is known that when the water flows from the soil to the porous plate changes in the hydrostatic pressure distribution occur. These modifications can affect the quality of data from the sample in regions close to the hydraulic contact associated with the interface between the soil sample bottom and the porous plate or sand (Alagna et al., 2016).

Imaging techniques such as X-ray Computed micro-Tomography offer great potential as a tool to visualize and subsequently better understand how changes in the soil pore structure might arise from W-D and thus their impact on the water retention curve. X-ray microtomography is a non-invasive and non-destructive technique that allows the study of morphological properties of the structure of the soil (Peth et al., 2008, Smet et al., 2017, Cássaro et al., 2017, Galdos et al., 2019, Pires et al., 2019). X-ray microtomography has been utilized for the analysis of soils since the 1980s (Petrovic et al., 1982). The ability to undertake three-dimensional (3D) analysis allows the evaluation of several soil structural properties such as porosity, number of pores, pore size, pore shape, fractal dimension, anisotropy, connectivity and tortuosity (Luo et al., 2010, Garbout et al., 2013, Dal Ferro et al., 2014, Borges et al., 2018, Ferreira et al., 2019, Diel et al., 2019).

Microtomography can provide important insights into how W-D affects soil pore structure at the microscale. Ma et al. (2015) analyzed changes in soil structure caused by W-D through synchrotron-based X-ray microtomography. They observed significant alterations in the soil porosity, pores >100 µm and in the fraction of elongated pores. Helliwell et al. (2017) observed significant changes in the soil structure in repacked cores after a single wetting and drying event, though further W-D had little impact. Further studies that evaluate modifications in soil pore structure in 3D at micrometric scale are scarce. Conversely, many studies have analyzed the effect of W-D in soil pore structure in two-dimensions (2D) (Sartori et al., 1985, Pagliai et al., 1987, Pires et al., 2008b, Rasa et al., 2012). However, 2-D images of pore structure only provide information about the area, perimeter, diameter, arrangement and size distribution of pores, which fails to account for the true heterogeneity of the soil structure (Bouma et al., 1977).

The aim of this study was to verify how alternations of W-D modify the soil pore structure morphological properties. Two sample volumes were analyzed: the first comprised almost the whole sample and the second, a small region close to the bottom of the sample. We hypothesized that there would be changes in the morphological properties of the soil as a consequence of W-D that affects the region close to the bottom of the sample differently in relation to the whole sample.

## 2 Materials and methods

### 2.1 Experimental site and soil sampling

Soil samples were obtained from an experimental field under zero tillage at the soil research unit of the Agricultural Research Institute of Parana (IAPAR) in the city of Ponta Grossa, PR, Brazil (25°06′S, 50°10′W, 875 m above sea level). The soil was an Oxisol (Rhodic Hapludox) according to USDA soil taxonomy (Soil Survey Staff, 2013). The soil was classified as a clay texture with 17% sand, 30% silt and 53% clay. The particle density and the amount of C content evaluated were 2.41 g cm^−3^ and 60.7 g kg^−1^, respectively.

Soil sampling was carried out at the beginning of 2017 from the surface layer (0–10 cm) after corn harvest in the middle of the crop interrows to avoid possible effects of tractor wheel traffic (cleaning, plant seed and soil preparation operations) on the soil structure. Undisturbed samples were collected in steel cylinders (c. 5 cm high and c. 5 cm diameter), with the help of an Uhland sampler, for the microtomography (9 samples) and soil water retention curve (18 samples) analyses. Sampling was undertaken very carefully, in order to prevent soil compaction during extraction and handling. The choice of samples collected in cylinders for this study was due to their use for water retention curve measurements. Since the soil water content is very important at the sampling time, to minimize damage in the soil structure, samples were collected when soils were near their field capacity, about three days after a high intensity rainfall event.

### 2.2 Wetting and drying cycles (W-D) for microtomography analysis

Soil samples were saturated by the capillary rise method. The wetting (W) procedure consisted in soaking the samples in a tray with the water level just below the top of the steel cylinders. This procedure was kept over a period of 2 days to allow saturation of the sample and to avoid the presence of the entrapped air bubbles, which can cause slaking of soil aggregates (Klute, 1986). Samples were partially dried by submitting them to a pressure head of −60 cm of H_2_O on a suction table (Eijkelkamp 08.01 Sandbox for pF determination). After reaching the thermodynamic equilibrium, the samples were again saturated and submitted to a new suction application (new drying) to simulate a series of W-D. This wetting and drying procedure was exactly the same as that employed to evaluate soil water retention curves (Klute, 1986). Three treatments were investigated: 0 W-D, in which samples were not submitted to any wetting and drying cycle, 6 and 12 W-D cycles.

### 2.3 Soil water retention curve measurement

The wetting procedure to saturate the samples was exactly the same as that described in the previous section. Following the saturation, the samples were placed in contact with the porous media (sand) on the suction table. The samples were equilibrated in the pressure heads varying from −10 to −100 cm of H_2_O with intervals of 10 cm (Romano et al., 2002). After the thermodynamic equilibrium was reached (nearly 4–5 days for each sample) the moist soil mass was evaluated using a precision balance (0.01 g). The dry soil mass was obtained at the end of the water retention curve by oven drying for 48 h at 105 °C.

The experimental pairs of data obtained (soil water contents and pressure heads) were fitted using the mathematical model proposed by van Genuchten-Mualem equation (van Genuchten, 1980). The Excel solver based on the total sum of squares was used for fitting the experimental data. The soil water retention curve adjustments were obtained using the average values of soil water contents (n = 6) for each treatment studied. In order to check the quality of the water retention curve fitting, the root-mean-square error and the coefficient of determination (R^2^) were calculated. Relative differences (RD) were also obtained between the water retention curves in order to evaluate the effect of the different W-D on the soil pore structure.

### 2.4 Computed tomography

The soil samples were carefully extracted from the steel cylinders before the microtomographic analysis to avoid the influence of the cylinder in the flux of X-ray photons. Prior to the scanning, the samples were coated with paraffin wax to minimize potential movement during transport from Brazil to the UK. More details about paraffin wax coating were described by Pires et al. (2019). This procedure was carried out after the application of the cycles for each treatment. Before coating, the samples were partially dried at 40 °C until their mass became constant. Each soil sample was scanned using a G.E. V-Tomex-M X-ray Computed Tomography scanner (GE Measurement & Control Solutions, Wunstorf, Germany) at the Hounsfield Facility (University of Nottingham, Sutton Bonington Campus, UK).

The voltage, current and integration time adopted for the image acquisition process were 180 kV, 160 µA and 250 ms. A 0.1 mm Cu-filter was used to minimize beam-hardening effects. A total of 2520 projections were obtained per sample with a voxel resolution of 35 µm. The radiographs of each scan were reconstructed in 32 bit format in order to prevent compression of the greyscale histogram. The gray scale of all 16‐bit images was calibrated to values based on the brightest (Mineral) and darkest (Air) objects in all of samples and then a grey level value was set based on the calculation of 2,661 for air and of 47,092 for the brightest mineral (in 16-bit depth). However, despite taking great care it is not possible to eliminate all potential scanning artifacts.

After reconstruction, the images were imported into Volumetric Graphics StudioMAX® 2.0 and cropped to a cubic shape (ROI_W_) with 29.8 × 29.8 × 29.8 mm (850 × 850 × 850 voxels). ROI_W_ was selected a few centimeters from the edge of the samples to minimize any influence of the paraffin wax in the soil structure (Pires et al., 2019). Another region of interest (ROI_HC_) smaller than the first one was also evaluated. This smaller region sized 29.8 × 29.8 × 7.0 mm (850 × 850 × 200 voxels) was selected inside the largest one, 2.45 mm away from the bottom of the sample.

Although the great interest in selecting the ROI_HC_ was to analyze the effect of W-D cycles in the region of hydraulic contact; unfortunately, it was impossible to select the exact region of the sample in which hydraulic contact with the sandbox occurs. The main reasons for that were the sample coating procedures, the irregularities in sample shape in this region and imaging artifacts at the edge of samples.

The original grey-level X-ray microtomographic images were processed using ImageJ 1.42 software (Rasband, 2007). An *unsharp mask* procedure with 1 voxel standard deviation and weighing 0.8 was applied to enhance the edge contrast. The segmentation process was based on the nonparametric and unsupervised Otsu method for thresholding (Otsu, 1979). The *remove outlier* tool with a 0.75 radius was applied to the images after segmentation. This process resulted in a binary image, in which pores and solids were represented by white and black pixels.

For the assessment of 3D soil structure, pores were classified according to their shape and size distribution. For the shape classification, geometrical parameters known as major, intermediate and minor axes of the ellipsoids that represent each pore were determined using 3D measuring techniques. These parameters were measured using the *Particle Analyser* tool in the ImageJ. Isolated pores <9 voxels were removed from the porous fraction of the images for the analyses of pore shape distribution to avoid potential dubious features from unresolved voxels (Jefferies et al., 2014).

The soil pores which allowed the measurement of the three main axes were classified according to the terminology suggested by Zingg (1935). The relation between the ratio of the intermediate by the major (Int./Maj.) axis and the ratio of the minor by the intermediate (Min./Int.) axis allows pore classification based on shape. Therefore, the pore shapes were classified as: Equant (EQ), Prolate (PR), Oblate (OB), and Triaxial (TR) (Pires et al., 2017).

The image-derived porosity and number of disconnected pores were calculated considering all resolvable pores. In this study, the term porosity refers to soil macropores only. The 3D pore size distribution was determined based on the volume of pores classified in different logarithmic volume intervals: 0.0001–0.01; 0.01–0.1; 0.1–1; 1–10; and >10 mm^3^.

The X-ray microtomographic images were also analyzed in terms of tortuosity of the pore network using the Osteoimage software (Roque et al., 2009). The tortuosity, which is geometrically defined by the ratio between the geodesic distance between two connected points and the Euclidean distance between these two points, was calculated through the geodesic reconstruction algorithm (Roque et al., 2012). The characteristic of Euler-Poincaré was utilized to estimate the degree of connectivity, which represents one of the Minkowski functions and a topological measure used for describing the connectivity of spatial structures (Vogel and Roth, 2001, Vogel et al., 2010, Katuwal et al., 2015). This parameter is related to the number of isolated parts minus the connectivity of an object (Thurston, 1997). Based on Euler-Poincaré values, the Euler-Poincaré per sample volume was evaluated. The Euler-Poincaré number is an indicator of how well connected a pore network is: the smaller (more negative) it is, the higher the pore connectivity is (Roque et al., 2009). The degree of anisotropy, which gives the preferred orientation of pores, was determined in 3D by using the BoneJ plugin (Doube et al., 2010). The pore volume interconnectedness was characterized by network properties. The *3D skeletonize* plugin (ImageJ) was applied to reduce iteratively the diameter of pores until only a skeleton was obtained. Parameters such as number of junctions and number of branches were measured using the ImageJ plugin *analyse skeleton*.

### 2.5 Statistical analysis

The data obtained via image analysis and water retention curve were submitted to Shapiro-Wilk and Bartlett tests to verify normality and homoscedasticity, respectively. When pre-suppositions had been verified (*p* > 0.05), since this is a nonparametric study, orthogonal contrasts between ROIs for each W-D and among W-D cycles for each ROI were employed. To obtain the significance (*p* ≤ 0.05) of the orthogonal contrast the Student *t*-test was applied. Simple linear correlation was performed by analyzing the Pearson’s correlation coefficients. All data were analyzed using the software R, version 3.6.1 (R Core Team, 2018).

## 3 Results and discussion

### 3.1 Morphological properties of porous system

**Fig. 1:**
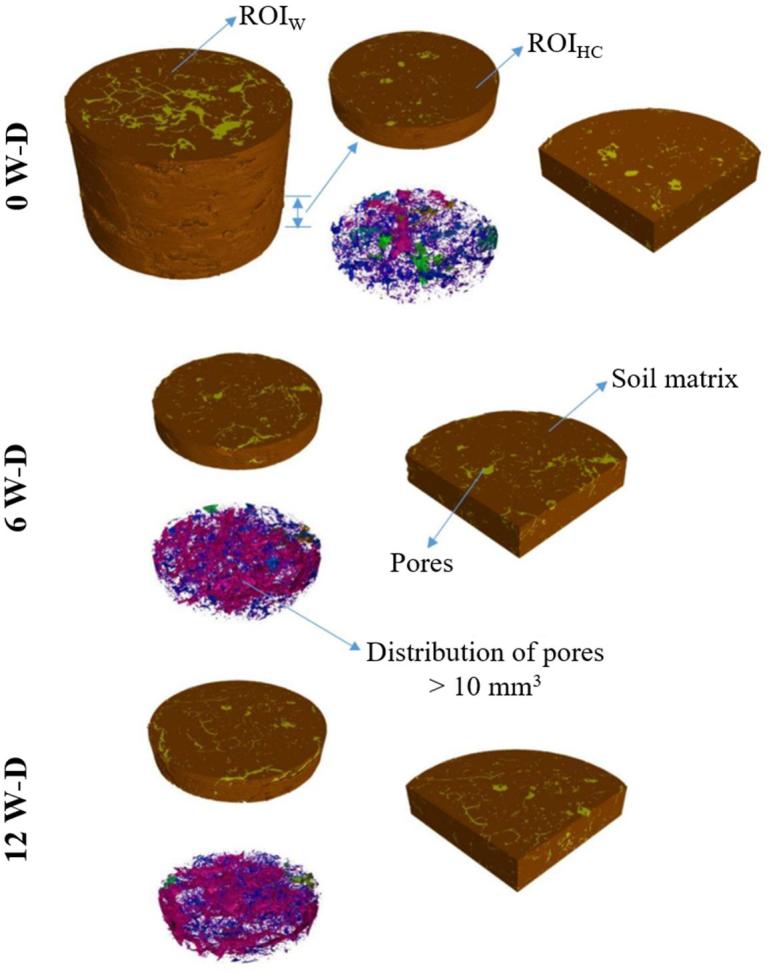
3D visualization of the soil samples before (0) and after the application of 6 and 12 wetting and drying (W-D) cycles. ROI_W_: whole region of interest. ROI_HC_: region of interest close to the bottom of the sample.

**Fig. 2:**
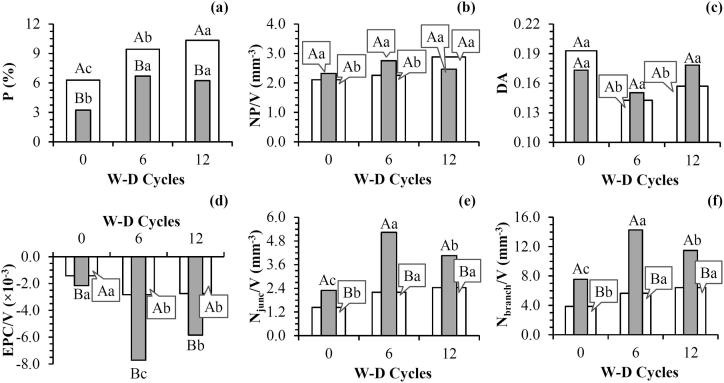
Morphological properties of the soil porous system before (0) and after the application of 6 and 12 wetting and drying (W-D) cycles: (a) Image-derived porosity (P); (b) Number of pores (NP); (c) Degree of anisotropy (DA); (d) Pore connectivity (EPC/V); (e) Number of junctions (N_junc_); (f) Number of branches (N_branch_). ROI_W_ (): whole region of interest. ROI_HC_ (): region of interest close to the bottom of the sample. Means followed by the same upper case letters between ROI_W_ and ROI_HC_ and same lowercase between W-D cycles did not differ from each other by t-Student test (*p* ≤ 0.05). n = 3 (number of samples analyzed for each treatment).

The porosity analyzed for the whole sample (ROI_W_) showed differences between 6 and 12 W-D cycles, as well as in relation to 0 W-D. However, for the region closer to the hydraulic contact (ROI_HC_), the action of 6 and 12 W-D cycles increased porosity in relation to control but did not differ from each other (Fig. 1). Soil porosity was also lower in ROI_HC_ in comparison to ROI_W_ for all W-D cycles, which means there were differences in the pore distributions inside the samples (Fig. 2a). The lower image-derived porosity for the lower portion of the samples may have been influenced by the procedures utilized for collecting samples in volumetric rings; as regions close to the walls of the cylinders can be subjected to stresses which damage the soil structure. Pires et al. (2004) has previously showed, through computed tomography imagery, the effects of different cylinder diameters in the soil structure due to sampling. As the lower region of the sample presented a decrease in its porosity, few wetting and drying cycles can provoke important changes in its structure. This was observed in our work for ROI_HC_, when 6 W-D cycles caused the most important changes in the soil structure for this region.

The number of pores increased after the application of 12 W-D cycles in relation to 0 and 6 W-D only for ROI_W_, while for ROI_HC_ no effects of W-D cycles were observed (Fig. 2b). We also noticed that the number of pores did not differ between ROI_W_ and ROI_HC_ for all W-D cycles analyzed. However, soil pore structure changes as shown by the porosity increase were not influenced by the increase in the number of pores after W-D cycles mainly for ROI_HC_ (Table 1).

**Table 1:** Pearson’s correlation coefficients between the morphological properties of the soil porous architecture for the whole region of interest (ROI_W_) and the region of interest close to bottom of the sample (ROI_HC_).

Variables	ROI_HC_									
	P	NP/V	DA	EPC/V	N_junc_/V	N_branch_/V	τ_average_	τ_x direction_	τ_y direction_	τ_z direction_
*Morphological properties*										
P	1.00									
NP/V	0.44	1.00								
DA	−0.17	−0.80*	1.00							
EPC/V	−0.95*	−0.51	0.32	1.00						
N_junc_/V	0.92*	0.44	−0.24	−0.98*	1.00					
N_branch_/V	0.95*	0.57	−0.35	−0.98*	0.97*	1.00				
τ_average_	−0.91*	−0.34	0.07	0.87*	−0.83*	−0.33*	1.00			
τ_x direction_	−0.95*	−0.41	0.13	0.87*	−0.83*	−0.86*	0.98*	1.00		
τ_y direction_	−0.46	−0.01	−0.43	0.24	−0.18	−0.28	0.59	0.63*	1.00	
τ_z direction_	**−0.86**	−0.60	0.32	0.90*	−0.87*	−0.88*	0.90*	0.91*	0.40	1.00
*Pore size distribution*										
VP_0.0001-0.01 mm_^3^	−0.96*	−0.33	0.08	0.90*	−0.88*	−0.86*	0.95*	0.96*	0.49	0.87*
VP_0.01-0.1 mm_^3^	−0.96*	−0.40	0.16	0.91*	−0.88*	−0.87*	0.95*	0.96*	0.48	0.90*
VP_0.1-1 mm_^3^	−0.97*	−0.38	0.13	0.91*	−0.88*	−0.88*	0.90*	0.93*	0.46	0.84*
VP_1-10 mm_^3^	−0.95*	−0.46	0.20	0.94*	−0.91*	−0.89*	0.89*	0.90*	0.36	0.89*
VP_>10 mm_^3^	0.96*	0.36	−0.09	−0.92*	0.90*	0.89*	−0.90*	−0.92*	−0.45	−0.87*
*Pore shape distribution*										
VP_Eq_	−0.24	0.01	−0.07	−0.02	0.06	0.02	0.29	0.37	0.51	0.09
VP_Pr_	−0.25	−0.26	0.39	0.45	−0.47	−0.48	0.04	0.02	−0.48	0.12
VP_Ob_	0.31	−0.38	0.54	−0.15	0.12	0.11	−0.47	−0.39	−0.56	−0.10
VP_Tr_	0.31	0.80*	−0.83*	−0.33	0.30	0.44	−0.11	−0.24	0.15	−0.29

	ROI_W_									

P	NP/V	DA	EPC/V	N_junc_/V	N_branch_/V	τ_average_	τ_x direction_	τ_y direction_	τ_z direction_

*Morphological properties*										
P	1.00									
NP/V	0.60	1.00								
DA	−0.77*	−0.50	1.00							
EPC/V	−0.92*	−0.46	0.84*	1.00						
N_junc_/V	0.86*	0.66*	−0.80*	−0.89*	1.00					
N_branch_/V	0.91*	0.62	−0.74*	−0.87*	0.95*	1.00				
τ_average_	−0.75*	−0.79*	0.75*	0.71*	−0.67*	−0.67*	1.00			
τ_x direction_	−0.85*	−0.73*	0.81*	0.89*	−0.90*	−0.86*	0.89*	1.00		
τ_y direction_	−0.70*	−0.69*	0.69*	0.63	−0.60	−0.59	0.87*	0.79*	1.00	
τ_z direction_	−0.74*	−0.77*	0.72*	0.67*	−0.59	−0.55	0.94*	0.81*	0.81*	1.00
*Pore size distribution*										
VP_0.0001-0.01 mm_^3^	−0.85*	−0.38	0.80*	0.94*	−0.71*	−0.71*	0.77*	0.84*	0.69*	0.74*
VP_0.01-0.1 mm_^3^	−0.88*	−0.51	0.81*	0.90*	−0.74*	−0.76*	0.84*	0.88*	0.85*	0.77*
VP_0.1-1 mm_^3^	−0.95*	−0.59	0.88*	0.98*	−0.88*	−0.88*	0.81*	0.92*	0.75*	0.77*
VP_1-10 mm_^3^	−0.96*	−0.55	0.88*	0.98*	−0.89*	−0.89*	0.75*	0.88*	0.69*	0.73*
VP_>10 mm_^3^	0.90*	0.57	−0.87*	−0.86*	0.72*	0.77*	−0.88*	−0.84*	−0.82*	−0.85*
*Pore shape distribution*										
VP_Eq_	0.80*	0.53	−0.83*	−0.78*	0.71*	0.74*	−0.82*	−0.82*	−0.92*	−0.71*
VP_Pr_	0.76*	0.39	−0.60	−0.61	0.45	0.62	−0.73*	−0.60	−0.74*	−0.64
VP_Ob_	0.70*	−0.28	0.52	0.54	−0.46	−0.67*	0.56	0.51	0.62	0.40
VP_Tr_	−0.61	−0.35	0.58	0.70*	−0.58	−0.47	0.52	0.65*	0.74*	0.54

P = Image-derived porosity; NP = Number of pores; DA = Degree of anisotropy; EPC/V = Pore connectivity; N_junc_ = Number of junctions; N_branch_ = Number of branches; τ_average_ = Average tortuosity; τ_x_, τ_y_ and τ_z_ = Tortuosity in the directions x, y and z, respectively; VP_0.0001-0.01 mm_^3^, VP_0.01-0.1 mm_^3^; VP_0.1-1 mm_^3^; VP_1-10 mm_^3^ and VP_>10 mm_^3^ = Volume of pores ranging from 0.0001 to 0.01 mm^3^, 0.01 to 0.1 mm^3^, 0.1 to 1 mm^3^, 1 to 10 mm^3^ and >10 mm^3^, respectively; VP_Eq_, VP_Pr_, VP_Ob_ and VP_Tr_ = Volume of equant, prolate, oblate and triaxial shaped pores. *p ≤ 0.05. n = 3 (number of samples analyzed for each treatment).

The application of W-D cycles can provoke swelling and shrinkage processes in the soil volume, which cause tension forces between aggregates. The action of these forces can reduce soil porosity when the force is directed from the border to the center of the aggregates, which takes place during sample drying. Schlüter et al. (2016) observed that the soil deformation, as consequence of shrinkage, occurs in any drying process for swelling clay minerals. According to these authors the capillary forces that pull unconsolidated grains close together can also cause changes in soil structure in drying processes. When the soil is submitted to wetting, the force follows the opposite direction from the center to the borders of the aggregates, which increases soil porosity (Peng et al., 2007, Bodner et al., 2013). As the samples may have been submitted to some damage during sampling, this may also help to explain the differences observed between regions of interest (ROI_W_ and ROI_HC_). The possible compaction induced by sampling in specific regions of the sample has higher capacity to recover the structure towards higher porosities than in the case of non-compacted samples.

Pore architecture modifications due to repeated W-D cycles have been described by several authors with potential reasons for this identified as a consequence of internal forces, including air entrapment and expansion between aggregates, natural reconsolidation of aggregates, aggregate fragmentation and generation of soil cracks (Tessier et al., 1990, Hussein and Adey, 1995, Li et al., 2004, Tang and Shi, 2011, Diel et al., 2019). As a consequence, those authors reported the main modifications in the soil pore structure as a function of W-D cycles usually occur in the size and shape of aggregates and pores, porosity, pore orientation and pore connectivity (Pardini et al., 1996, Hussein and Adey, 1998, Peng et al., 2007, Tracy et al., 2015, Zhang et al., 2018).

During W-D cycles, the pressure caused by the water movement until the hydraulic equilibrium is reached by the samples can cause the removal of clay particles from the surface of soil aggregates, which might reduce their stability (Czyż and Dexter, 2015, Ma et al., 2015). The dispersed particles could: (i) migrate to ROI_HC_ sealing the pores located at the bottom of the sample in contact to the sandbox decreasing soil porosity (Zhang et al., 2014, Périard et al., 2016) or (ii) be removed from the samples to the sandbox (Reynolds and Topp, 2006, Pires et al., 2011). The latter, which simulates the eluviation/illuviation processes in the soil profile, would be dependent on the pressure head applied to the sample as well as the characteristics of the dispersed clay (Czyż and Dexter, 2015).

However, it is important to mention that different soil types are likely to present different results than the observed in our study. Clay minerals present in the soil can differ considerably in several properties such as specific surface, shape, volume, etc., which will influence the clay particle dynamics under wetting and drying (Jury and Horton, 2004). For example, we would expect less severe changes in the soil structure due to the W-D cycles for sandy in the comparison to clayey soils, as investigated in our work. This is related to the main minerals that compose the sandy soils and their capacity to pack and hold together the particles in aggregate form, which will influence the production of intra and inter-aggregate pores (Hillel, 2004).

**Fig. 3:**
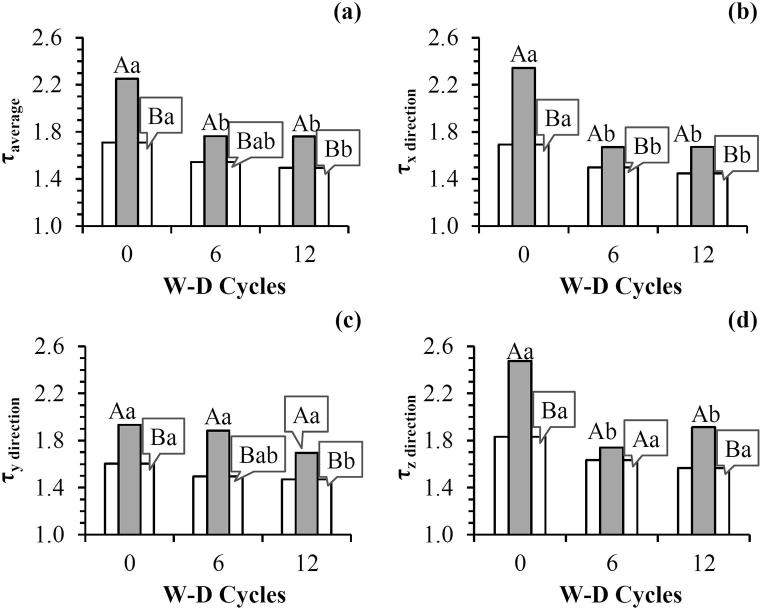
(a) Average tortuosity (τ); (b) Tortuosity in the x-direction; (c) Tortuosity in the y-direction; (d) Tortuosity in the z-direction of soil pores before (0) and after the application of 6 and 12 wetting and drying (W-D) cycles. ROI_W_ (): whole region of interest. ROI_HC_ (): region of interest close to the bottom of the sample. Means followed by the same upper case letters between ROI_W_ and ROI_HC_ and same lowercase between W-D cycles did not differ from each other by t-Student test (*p* ≤ 0.05). n = 3 (number of samples analyzed for each treatment).

Soil pore structure was not affected by the concentration of dispersed clay in ROI_HC_, since decreases in soil porosity were not observed after the application of W-D cycles (Fig. 2a). The results show the forces acting on drying probably overcome those acting on wetting (Bodner et al., 2013). The decrease in tortuosity (Fig. 3 and Table 1), with the application of W-D cycles can be considered as evidence of this hypothesis, because an interconnection of the pores can be related to more continuous flow channels (Peth et al., 2008).

The soil pore architecture modifications due to W-D cycles did not cause heterogeneities in the pore distributions in ROI_HC_ as verified by the anisotropy results. In relation to ROI_W_, both 6 and 12 W-D cycles reduced the degree of anisotropy in comparison to the samples not submitted to W-D cycles. Considering the W-D cycles, the different regions studied did not differ from each other in terms of anisotropy (Fig. 2c). Similar results were found by Piccoli et al. (2019), who found that the anisotropy of the soil is not affected by the sample volume; although tillage processes can affect significantly this property (Piccoli et al., 2017).

Pore connectivity increased after 6 and 12 W-D cycles for ROI_HC_ and ROI_W_. ROI_HC_ had a higher pore connectivity than ROI_W_. The soil pore structure in ROI_HC_ was greatly influenced by W-D cycles in terms of pore connectivity (Fig. 2d). The increase in pore connectivity for ROI_HC_ was also followed by an increase in the number of junctions and branches of pores induced by the cycles in relation to 0 W-D. This result could also help to explain the increase in soil porosity following W-D cycles. The number of junctions and branches was also affected by the number of W-D cycles for ROI_W_ (Fig. 2e, f) with lower values compared to ROI_HC_. This is an indication of a more complex soil structure in the region close to the bottom of the sample in relation to the whole sample as a result of reorganization of some kind.

The increase in the porosity influenced positively the number of junctions and branches and negatively the pore connectivity for ROI_W_ and ROI_HC_ (Table 1). However, pore connectivity was not affected by the increase in the number of pores for ROI_W_ and ROI_HC_, although for ROI_W_ the number of junctions were positively correlated to the number of pores. This result could be explained by the larger volume of sample analyzed for ROI_W_.

The increase in pore connectivity was accompanied by a decrease in the number of junctions and branches mainly for ROI_HC_, which was more susceptible to changes in relation to the whole sample (Table 1). For ROI_W_ the increase in the porosity with the W-D cycles was followed by a decrease in anisotropy and an increase in pore connectivity. For ROI_HC_ no significant correlations for anisotropy were observed.

The average tortuosity and the tortuosity in the different directions (x, y, and z) decreased for both ROIs with W-D cycles (Fig. 3). The region close to the bottom of the samples was characterized by a higher tortuosity than the whole sample (ROI_W_). This result was independent on the W-D cycles. We expected an increase in tortuosity with the cycles due to the increase observed in the number of junctions and branches. However, this was not observed in our study.

The decrease in the average tortuosity was followed by an increase in pore connectivity and in the number of junctions and branches for ROI_W_ and ROI_HC_ (Table 1). These results indicate that more aligned pores were characterized by a greater number of connected pores, mainly for ROI_HC_. This is interesting because these two morphological properties are known to influence water movement (Sayem and Kong, 2016). Since the water movement from the bottom of the sample to the sandbox is greatly dependent on the soil pore structure, changes in pore connectivity and tortuosity can have important influence in the soil water retention curve evaluation due to W-D cycles (Fig. 2d and 3) (Pires et al., 2008a, Rafraf et al., 2016). Dörner and Horn (2006) pointed out that even when small changes in soil porosity are observed, significant modifications in pore continuity and geometry can present great influence on soil hydraulic properties.

### 3.2 Pore shape and size distributions

**Fig. 4:**
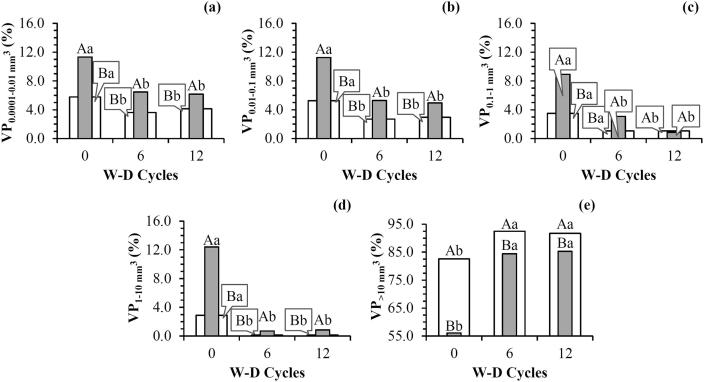
Pore size distribution based on volume before (0) and after the application of 6 and 12 wetting and drying (W-D) cycles: (a) Volume of pores (VP) between 0.0001 and 0.01 mm^3^ (VP_0.0001-0.01_); (b) VP between 0.01 and 0.1 mm^3^ (VP_0.01-0.1_); (c) Volume of pores between 0.1 and 1 mm^3^ (VP_0.1-1_). (d) VP between 1 and 10 mm^3^ (VP_1-10_); (e) VP > 10 mm^3^ (VP_>10_). ROI_W_ (): whole region of interest. ROI_HC_ (): region of interest close to the bottom of the sample. Means followed by the same upper case letters between ROI_W_ and ROI_HC_ and same lowercase between W-D cycles did not differ from each other by t-Student test (*p* ≤ 0.05). n = 3 (number of samples analyzed for each treatment).

The distribution of pore sizes was affected by the W-D cycles for ROI_W_ and ROI_HC_ (Fig. 4). Volume of pores presented a significant decrease between 0.0001 and 0.01, 0.01–0.1, 0.1–1 and 1–10 mm^3^ pore size classes after 6 and 12 W-D cycles in comparison to the control treatment (Fig. 4a to 4d). For the different ROIs analyzed the same behavior was noticed between 0, 6 and 12 W-D cycles, except for 12 W-D cycles for pores with sizes between 0.1 and 1 mm^3^ (Fig. 4c). The influence of these pore classes in soil porosity was greater for ROI_HC_ in comparison to ROI_W_ (Fig. 4a, b).

For the largest pores (>10 mm^3^), the ROI_W_ volume of pores was significantly larger than that of ROI_HC_ for 0, 6 and 12 W-D cycles (Fig. 4e). Volume of pores also increased with the application of W-D cycles for the largest pore sizes for ROI_HC_ and ROI_W_. This result explains the increase in soil porosity (Table 1), which is related to an increase in the number of pores joined together (Fig. 1).

Several authors have reported increases in the volume of large pores in clayey soils following W-D cycles, as a consequence of textural effects and interlayer swelling at microscopic and macroscopic scales (Sartori et al., 1985, Pires et al., 2008b, Zemenu et al., 2009, Peng et al., 2007, Ma et al., 2015). Bodner et al. (2013) demonstrated that the intensity of W-D increases the macroporosity for soils with more stable structures, such as those found under zero-tillage management. The increase in the volume of large pores will certainly impact water retention for high pressure heads (less negative) due to lower capillary forces caused by larger pores (Périard et al., 2016).

Significant correlations were found between the distribution of pore sizes and the micromorphological properties studied as a function of W-D cycles, which showed different behavior between ROI_HC_ and ROI_W_ (Table 1). The increase in soil porosity and volume of pores >10 mm^3^ (positive correlation) produced a more heterogeneous soil structure, which was confirmed by the results of the number of junctions and branches. A high density of branches and junctions is related to an extensive, well-connected and complex pore network (Peth et al., 2008, Munkholm et al., 2012). However, the tortuosity was the converse (Table 1), which can be explained by the great influence of larger macropores to soil porosity. Samples presenting a high volume of pores >10 mm^3^ are normally characterized by a large number of junctions and branches when all the pores from 3D images are analyzed (Garbout et al., 2013). According to our results (Table 1), we reinforce the importance of these changes mainly when occurring in the region close to the bottom of the sample (ROI_HC_).

**Fig. 5:**
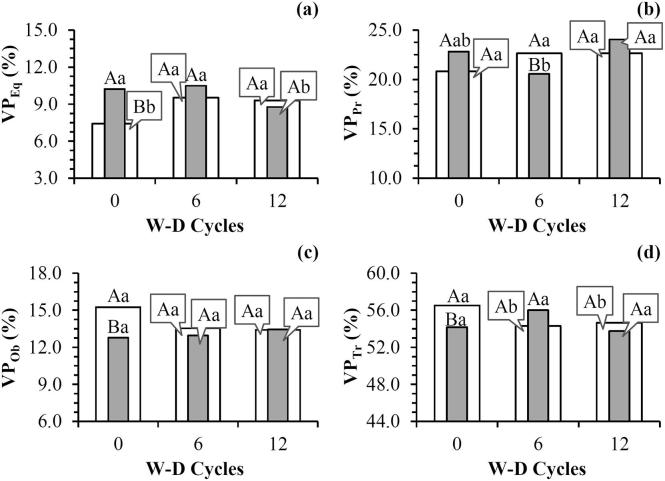
Pore distribution based on shape before (0) and after the application of 6 and 12 wetting and drying (W-D) cycles: (a) Equant shaped volume of pores (VP_Eq_); (b) Prolate shaped volume of pores (VP_Pr_); (c) Oblate shaped volume of pores (VP_Ob_); (d) Triaxial shaped volume of pores (VP_Tr_). ROI_W_ (): whole region of interest. ROI_HC_ (): region of interest close to the bottom of the sample. Means followed by the same upper case letters between ROI_W_ and ROI_HC_ and same lowercase between W-D cycles did not differ from each other by t-Student test (*p* ≤ 0.05). n = 3 (number of samples analyzed for each treatment).

The distribution of pores in terms of shape presented differences between ROIs with the W-D cycles for the equant and triaxial shaped pores (Fig. 5). The cycles caused an increase in the equant shaped volume of pores for ROI_W,_ while the opposite was observed for ROI_HC_ (Fig. 5a). For the triaxial shaped pores, a decrease in these pore types was recorded for ROI_W_ (Fig. 5d). Few significant correlations were measured between the distribution of pores in terms of shape and the micromorphological properties investigated, mainly for ROI_HC_ (Table 1). For ROI_W_, pore shape was related with pore connectivity and tortuosity in the x and z directions. The increase in the equant and prolate shaped pores and the decrease in triaxial shaped pores influence the volume of pores that are related to an increase in pore connectivity. This was also observed for the average tortuosity and the tortuosity in the different directions. Only the triaxial shaped volume of pores variation was not related to tortuosity (Table 1). Differences in the pore shape distribution are important because there is a close relation between pore shape and water retention and movement in the soil (Pagliai and Vignozzi, 2002, Yoon et al., 2007).

However, it is important to mention that >60% of the pores were not classified as they had a complex shape that is probably related to the junctions of the pores following the application of the W-D cycles.

### 3.3 Soil water retention

The water retention curves showed the W-D cycles treatment influence in the soil structure. In our study computed tomography was used to reveal the structural arrangements of the soil sample following W-D treatment and not compare the pore size distribution with the water retention curve. The pressure head range was selected according to the resolution of the microtomographic images. However, the water retention data allowed us also to analyze pores smaller than the resolution obtained by computed tomography imagery, *i.e.*, textural pores.

**Fig. 6:**
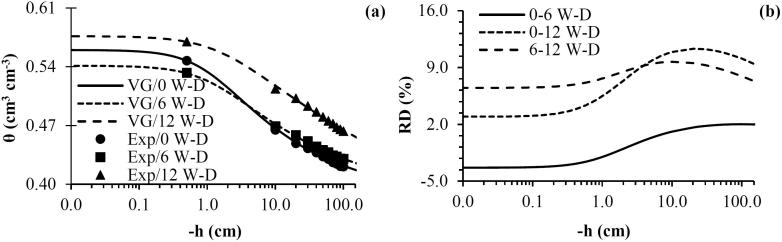
(a) Water retention curve (SWRC); (b) SWRC relative differences before (0) and after the application of 6 and 12 wetting and drying (W-D) cycles. VG: van Genuchten-Mualem mathematical model. Exp: Experimental data. n = 6 (number of samples analyzed for each treatment).

The soil water retention curve was most influenced by the application of 12 W-D cycles as observed by the van Genuchten-Mualem model parameters (Table 2). Higher water retention was found for the range of pressure heads analyzed with 12 W-D cycles (Fig. 6a). This implies that the application of 12 W-D caused an increase in pores from textural to structural pore size ranges (from 30 to >100 µm equivalent cylindrical diameter), *i.e.*, medium to coarse pores. This result is partially supported by the computed tomography data (Fig. 4). The application of W-D cycles can promote changes in fine matrix pores especially when clayey soils are dried due to the susceptibility of the soil to swelling and shrinkage. W-D cycles can also cause changes in the largest pores as in our study which helps to explain the results for the water retention for 12 W-D cycles (Fig. 4e).

**Table 2:** Soil water retention curve parameters from the van Genuchten (1980) mathematical model before (0) and after the application of 6 and 12 wetting and drying (W-D) cycles.

W-D cycles	θ_s_	θ_r_	α	n	m	R^2^
	cm^3^ cm^−3^		cm^−1^			
0	0.560	0.382	0.914	1.329	0.248	0.99
6	0.541	0.378	0.812	1.258	0.205	0.99
12	0.576	0.307	0.579	1.132	0.117	0.99

n = 6 (number of samples analyzed for each treatment).

**Table 3:** Pearson’s correlation coefficients between the morphological attributes of the soil porous architecture for the whole region of interest (ROI_W_) and soil water retention curve parameters based on the van Genuchten (1980) mathematical model.

Variables	van Genuchten (1980) parameters			
	θ_s_	θ_r_	α	n
*Morphological properties*				
P	0.16	−0.69*	−0.84*	−0.87*
NP/V	0.58	−0.80*	−0.80*	−0.79*
DA	0.28	0.26	0.47	0.51
EPC/V	0.10	−0.48	0.68*	0.73*
N_junc_/V	0.16	−0.65*	−0.79*	−0.82*
N_branch_/V	0.23	−0.71*	−0.83*	−0.85*
τ_average_	−0.12	0.53	0.64	0.66*
τ_x direction_	−0.14	0.62	0.76*	0.79*
τ_y direction_	−0.09	0.47	0.58	0.61
τ_z direction_	−0.13	0.53	0.64	0.66*
*Pore size distribution*				
VP_0.0001-0.01 mm_^3^	0.25	0.32	0.53	0.57
VP_0.01-0.1 mm_^3^	0.13	0.42	0.61	0.65*
VP_0.1-1 mm_^3^	0.05	0.53	0.72*	0.76*
VP_1-10 mm_^3^	0.05	0.54	0.73*	0.77*
VP_>10 mm_^3^	−0.11	−0.43	−0.62	−0.66*
*Pore shape distribution*				
VP_Eq_	−0.12	−0.37	−0.54	−0.58
VP_Pr_	−0.03	0.35	0.48	−0.51
VP_Ob_	−0.01	0.34	0.45	0.47
VP_Tr_	0.14	0.29	0.46	0.49

P = Image-derived porosity; NP = Number of pores; DA = Degree of anisotropy; EPC/V = Pore connectivity; N_junc_ = Number of junctions; N_branch_ = Number of branches; τ_average_ = Average tortuosity; τ_x_, τ_y_ and τ_z_ = Tortuosity in the directions x, y and z, respectively; VP_0.0001-0.01 mm_^3^, VP_0.01-0.1 mm_^3^; VP_0.1-1 mm_^3^; VP_1-10 mm_^3^ and VP_>10 mm_^3^ = Volume of pores ranging from 0.0001 to 0.01 mm^3^, 0.01 to 0.1 mm^3^, 0.1 to 1 mm^3^, 1 to 10 mm^3^ and > 10 mm^3^, respectively; VP_Eq_, VP_Pr_, VP_Ob_ and VP_Tr_ = Volume of equant, prolate, oblate and triaxial shaped pores. *p ≤ 0.05. n = 3 (number of samples analyzed for each treatment for computed tomographic analysis). n = 6 (number of samples analyzed for each treatment for soil water retention curve).

Differences of around 10% were recorded between −5 to −100 cm when the samples were submitted to 12 W-D in relation to 0 W-D cycles (Fig. 6b). Nonetheless, the application of 6 W-D cycles did not appear to generate significant changes in the soil structure in relation to the control samples (0 W-D). This result was not expected considering the results of porosity and pore size distribution obtained via computed tomography imagery. However, it is important to note that the samples utilized for the water retention analysis were not the same as those used in computed tomography analysis; thus spatial variability could influence the results observed. Zhou et al. (2017) pointed out that it is hard to compare results from computed tomography and water retention due to the differences between methods and the soil pore range over different orders of scale.

The largest difference between 0 and 6 W-D cycles was around 2% for −20 to −100 cm (Fig. 6b). This means that only after the application of more than 6 W-D cycles, the soil under zero-tillage presented important modifications to its structure. This was confirmed by the comparison between 6 and 12 W-D cycles. The largest difference observed was around 9% for between −5 and −60 cm (Fig. 6b). Denef et al., 2001a, Denef et al., 2001b) reported the amount of large macroaggregates was reduced after the first W-D and after the second cycle they became stable and resistant to disintegration. Zhang et al. (2018) pointed out that depending on the experimental setup and the soil texture, a large number of W-D can be necessary to cause important changes in soil structure. Similar results were found by Pires et al. (2005) working with clay and sandy Brazilian soils.

To understand the dependence on changes in morphological properties of the soil pore structure and water retention, a correlation analyses were carried out between these properties and van Genuchten-Mualem fit parameters for ROI_W_ (Table 3) but only a few parameters presented any correlation. Possible explanations are: (i) the computed tomography analysis was not performed on the same samples of soil water retention curve evaluation as previously mentioned, (ii) the volume of analysis considered was not the same between the two techniques and (iii) the resolution limitation of computed tomography imaging used in this study only allows the evaluation of mainly pores classified as structural pores (Zhou et al., 2017).

The parameter α was inversely related to the soil porosity, number of pores, number of junctions and showed a positive correlation with pore connectivity, volume of pores between 0.1 and 1 and 1–10 mm^3^ and tortuosity in x-direction (Table 3). This parameter allow us to evaluate what happens with the large structural pores close to the water saturated region of the measured water retention curves (Bruand and Cousin, 1995). Smaller values of α are directly related to decreases in structural pores (Stange and Horn, 2005). We observed that the contribution of structural pores to the soil porosity was affected by W-D cycles, especially after 12 W-D cycles (Fig. 4). The parameter n was inversely related to the soil porosity, number of pores, number of junctions and branches and volume of pores >10 mm^3^; and positively related to the average tortuosity, the tortuosity in the x and z-directions, the volume of pores between 0.01–0.1, 0.1–1 and 1–10 mm^3^ (Table 3). This parameter indicates the difference in the amount of water retained between 0 and −100 cm which was small for 6 and 12 W-D, in the comparison to 0 W-D cycles. This result shows clear evidence of the effect of changes in the distribution of pore sizes with the application of W-D cycles (Fig. 4).

However, the fact that only few morphological properties correlated with the water retention van Genuchten-Mualem fitting parameters shows the difficult in trying to comparing soil physical properties from methods that consider measurements across different spatial scales. The limited number of soil samples investigated in this study may also contribute to the lack of correlations among the majority of the parameters analyzed.

## 4 Conclusions

Soil samples can exhibit distinct changes in their pore architecture structure, as well as water retention, as function of repeated W-D cycles. The soil close to the hydraulic contact with the sandbox as part of measurement of the water retention curve presented similar behavior to the rest of soil sample which was surprising as the pore connectivity and tortuosity measured by computed tomography imagery was greatly affected by W-D cycles for this region. The water movement in the soil towards the sandbox is greatly influenced by these two parameters, which would be expected to affect the representativeness of the water retention curve. The application of both 6 and 12 W-D cycles increased the image-derived soil porosity, volume of larger pores and pore connectivity in ROI_HC_. The tortuosity of the pore network was reduced with the application of W-D cycles, especially in ROI_HC_. When considering the water retention curve the differences were mainly observed in samples which were submitted to 12 W-D cycles, which had an increase in the amount of water retained for the structural and textural pores. Though, we note in this study, only a clay textured soil was considered in which the structural rearrangement following W-D is enhanced compared to a coarser textured soil such as predominantly sandy ones.

## Declaration of Competing Interest

The authors declare that they have no known competing financial interests or personal relationships that could have appeared to influence the work reported in this paper.
